# A CMOS IC-based multisite measuring system for stimulation and recording in neural preparations *in vitro*

**DOI:** 10.3389/fneng.2014.00039

**Published:** 2014-10-10

**Authors:** Takashi Tateno, Jun Nishikawa

**Affiliations:** ^1^Special Research Promotion Group, Graduate School of Frontier Biosciences, Osaka UniversityOsaka, Japan; ^2^Bioengineering and Bioinformatics, Graduate School of Information Science and Technology, Hokkaido UniversitySapporo, Japan

**Keywords:** bidirectional electrode, CMOS IC technology, low-noise amplifier, neural networks, noise suppressor

## Abstract

In this report, we describe the system integration of a complementary metal oxide semiconductor (CMOS) integrated circuit (IC) chip, capable of both stimulation and recording of neurons or neural tissues, to investigate electrical signal propagation within cellular networks *in vitro*. The overall system consisted of three major subunits: a 5.0 × 5.0 mm CMOS IC chip, a reconfigurable logic device (field-programmable gate array, FPGA), and a PC. To test the system, microelectrode arrays (MEAs) were used to extracellularly measure the activity of cultured rat cortical neurons and mouse cortical slices. The MEA had 64 bidirectional (stimulation and recording) electrodes. In addition, the CMOS IC chip was equipped with dedicated analog filters, amplification stages, and a stimulation buffer. Signals from the electrodes were sampled at 15.6 kHz with 16-bit resolution. The measured input-referred circuitry noise was 10.1 μ V root mean square (10 Hz to 100 kHz), which allowed reliable detection of neural signals ranging from several millivolts down to approximately 33 μ V_pp_. Experiments were performed involving the stimulation of neurons with several spatiotemporal patterns and the recording of the triggered activity. An advantage over current MEAs, as demonstrated by our experiments, includes the ability to stimulate (voltage stimulation, 5-bit resolution) spatiotemporal patterns in arbitrary subsets of electrodes. Furthermore, the fast stimulation reset mechanism allowed us to record neuronal signals from a stimulating electrode around 3 ms after stimulation. We demonstrate that the system can be directly applied to, for example, auditory neural prostheses in conjunction with an acoustic sensor and a sound processing system.

## Introduction

Multielectrode arrays (MEAs) allow researchers to stimulate and record multiple electrical signals at the single-cell level, and thus have become an indispensable tool in the study of neural network properties such as network formation (Maeda et al., 1995; Kamioka et al., 1996), network dynamics (Streit et al., 2001; Jimbo et al., 2003), and signal processing (Meister et al., 1994). An advantage of MEAs is that they can be arranged in planar structures using microfabrication technology, allowing for the investigation not only of neuronal cultures and brain slices *in vitro*, but also of animal tissues *in vivo* (Rubehn et al., 2009; Hampson et al., 2012; Hottowy et al., 2012). Several recent studies also reported the system integration of a complementary metal oxide semiconductor (CMOS) integrated circuit (IC) chip that is capable of both recording and stimulation of neurons or neural tissues (Hafizovic et al., 2007; Hottowy et al., 2008).

In particular, recent studies have reported the development of prototype bidirectional (recording and stimulation) systems *in vitro* and *in vivo* (Madhavan et al., 2006; Newman et al., 2012). However, in contrast to both modern MEA-based systems that are capable of recording complex spatiotemporal neural network activity patterns and to recent closed-loop electrophysiological systems (Rolston et al., 2010), the functionality of these CMOS IC-based systems with respect to the electrical stimulation of complex neuronal activity is very limited. The stimulation is usually restricted to a single electrode or several electrodes following the same stimulation protocol (Dabrowski et al., 2004). More sophisticated approaches include the definition of several independent stimulation signals that can be repeated by groups of electrodes (Charvet et al., 2010; Frey et al., 2010) or the fast switching of single-channel stimulation circuitry between electrodes (Wagenaar et al., 2004). In addition, neural stimulation is a key function performed by recent implantable medical devices for applications requiring many stimulation sites such as retinal prostheses for the blind and cochlear implants for the hearing disability. Such medical devices for the sensory neural prostheses need a completely flexible spatiotemporal stimulation to encode sensory information in the environment. However, although these CMOS IC-based systems can generate patterns of stimulation signals that are distributed in space and time, few of them exploit the potential of modern MEAs to elicit complex, arbitrarily defined activity patterns in a large number of neurons (Hafizovic et al., 2007).

An additional problem common to all systems aiming at simultaneous electrical stimulation and recording is stimulation artifacts. The electrical signals applied to activate neurons are sensed by all electrodes of the array as stimulus-related artifacts, with amplitudes several orders of magnitude larger than the amplitudes of the recorded action potentials. This can result in the saturation of the recording amplifier and makes the detection of the neuron response very difficult. Although the artifacts can be vastly reduced in magnitude by the optimization of the stimulation circuitry and experimental protocol (Jimbo et al., 2003; Brown et al., 2008; Frey et al., 2010), as well as by additional signal post-processing during data analysis (Wichmann, 2000; Wagenaar and Potter, 2002; Gnadt et al., 2003; Sekirnjak et al., 2006), most current CMOS IC-based MEA systems are unable to record neuronal responses on the stimulating electrode for at least 2 ms following the stimulus, and for at least half a millisecond on the nearby non-stimulating electrodes. In comparison, the delay of the elicited action potential in response to the stimulation pulse can be as short as 100 μ s (Sekirnjak et al., 2008) and the duration of the recorded pulse is on the order of a millisecond. Therefore, the detection and proper identification of fast neuronal responses with such systems are extremely difficult. To avoid large stimulation artifacts and to archive charge balancing, some design techniques for neural stimulators have been presented (Hafizovic et al., 2007; Rolston et al., 2009). However, there are few reports regarding application of CMOS IC-based MEA systems to brain slice preparation (David-Pur et al., 2014), although some studies have described possible applications in the future (Hutzler et al., 2006; Berdondini et al., 2009).

In this study, we report on the system integration of a CMOS IC chip that is capable of both stimulation and recording of neurons or neural tissues, permitting investigation of electrical signal propagation within cellular networks *in vitro*. In our system, the frequency range of recording signals is adjustable for specific targets, including action potentials and local field potentials (FPs). In addition, although stimulation in typical CMOS IC-based MEA systems has been strictly limited to simple pulse patterns and few electrodes, we describe an application-specific integrated circuit (ASIC) system designed for multisite electrical stimulation of neural tissue using standard MEAs. Our design is intended for applications in systems requiring simultaneous stimulation and recording of signals from various types of neural tissue, both *in vitro* (especially in brain slice preparations) and *in vivo*. The developed ASIC comprises 64 independent stimulation channels, any subset of which may be stimulated, and can generate almost arbitrarily defined mono/bi-polar voltage pulses below an amplitude of 2.5 V with 5-bit resolution. Each channel is also equipped with a stimulation artifact suppressor controlled in real time, which reduces the dead time of the system after each stimulation pulse. The system we developed can be used for conventional electrophysiological experiments *in vitro*, and the technology is also a foundation for future auditory neural prostheses applied to the auditory central nervous system.

## Materials and methods

### Total system description: recording and stimulation

**Figure 1:**
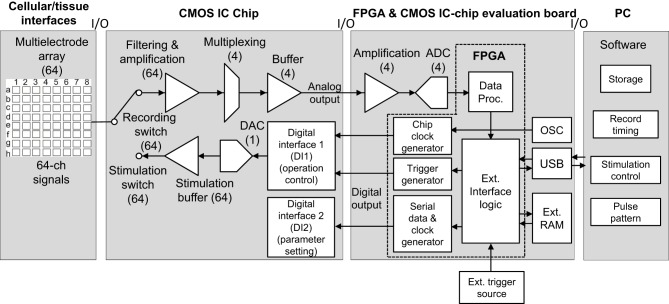
**System overview with respect to signal and data flow**. In this system, neural signals and recorded data are processed from left to right (cellular/tissue interfaces to a PC), while stimulation data propagate from right to left (a PC to cellular/tissue interfaces). In this study, the leftmost block labeled “Cellular/tissue interfaces” is specifically a planar MEA, but other types of multielectrode (e.g., penetrate electrode or surface electrode) array can be used. The second block labeled “CMOS IC chip” includes components in multiple realizations. In all blocks, numbers in brackets indicate the number of realizations per chip; e.g., 64 electrodes, 64 amplifiers, 4 buffers, and so on. The arrows indicate the direction of signals and data flow. In the FPGA and CMOS IC evaluation board block, “OSC” represents an oscillator. For the FPGA and CMOS IC evaluation board and PC software, see text and **Figures 3, 4**.

The total system, excluding cellular and/or tissue interfaces, includes three components: (1) the CMOS ASIC chip, (2) the FPGA board for the chip evaluation, and (3) the PC (Figure 1). In the ASIC chip, each of the 64 readout channels includes amplification and filter stages. The gain and filter settings of the stages can be programed digitally. Sixteen channels are then multiplexed and buffered by each of four amplifiers in the next stage, followed by analog-to-digital converter (ADC) processing at 15.6 kHz per channel, providing 16-bit resolution in the FPGA board. The data are subsequently transferred to a data processing unit in the FPGA. In contrast, two digital interfaces (DIs) in the chip decode commands that are used to define parameters of the stimulation and operation control (DI1) as well as those of signal amplitude and bias settings (DI2). To feed a voltage pulse series into electrodes, the system includes 64 sets of stimulation buffers and 5-bit digital-to-analog converters (DACs). The command decoder in the DI1 allows for specification of which DAC to use and configures the buffer in voltage mode.

### Signal processing circuitry

The signal processing circuitry was designed to meet the specification of extracellular (capacitive) recordings of neural cell activity. Neural signals usually have amplitudes of hundreds of microvolts and frequency ranges from approximately 100 Hz to 6 kHz (Najafi, 1994). Many CMOS IC-based neural amplifier designs have been reported in the literature (Harrison and Charles, 2003; Mohseni and Najafi, 2004; Olsson et al., 2005; Perelman and Ginosar, 2007; for review, Gosselin, 2011). Most amplifiers achieve a low input-referred noise of 5 μ V for bandwidths of 5–10 kHz. In particular, the use of a CMOS-bipolar pseudoresistor element as a high-resistance element and on-chip DC-coupling capacitors enables the amplifier to reject large DC offsets at electrode-tissue interfaces (Harrison and Charles, 2003) while allowing neural signals of interest to pass. Since the technique permits high-resistance elements to be implemented in a small area on-chip, large off-chip components are not needed. The amplifier reported by Harrison and Charles (2003) uses a standard wide-output swing operational transconductance amplifier (OTA) with capacitive feedback to realize a gain of approximately 40 dB, and presents design techniques that minimize the input-referred noise of the amplifier by operating some devices of the OTA in strong inversion to minimize their noise contributions. Although our amplifiers in this study use a similar technique, they can be configured for either recording neural spikes or local FPs by altering their bandwidth. Particularly, in usual extracellular recordings, offset and drift that occur at the electrode can be significantly larger than the signal amplitude. These effects need to be eliminated to permit amplification of the small neural signals. To this end, higher and lower corner frequencies were respectively used in low- and high-pass filters. The high-pass filter (HPF) has been filter with one of several adjustable corner frequencies (e.g., 0.10, 1.0, 10, or 100 Hz). Next, the signals pass the low-pass filter (LPF) with one of several tunable (reconfigurable) corner frequencies (e.g., 1.0, 2.5, 5.0, 7.5, and 10 kHz) to prevent high-frequency aliasing and to limit noise bandwidth. Finally, a buffer with larger bandwidth has been realized to further amplify the signal and to allow multiplexing (cf. Figure 1).

**Figure 2:**
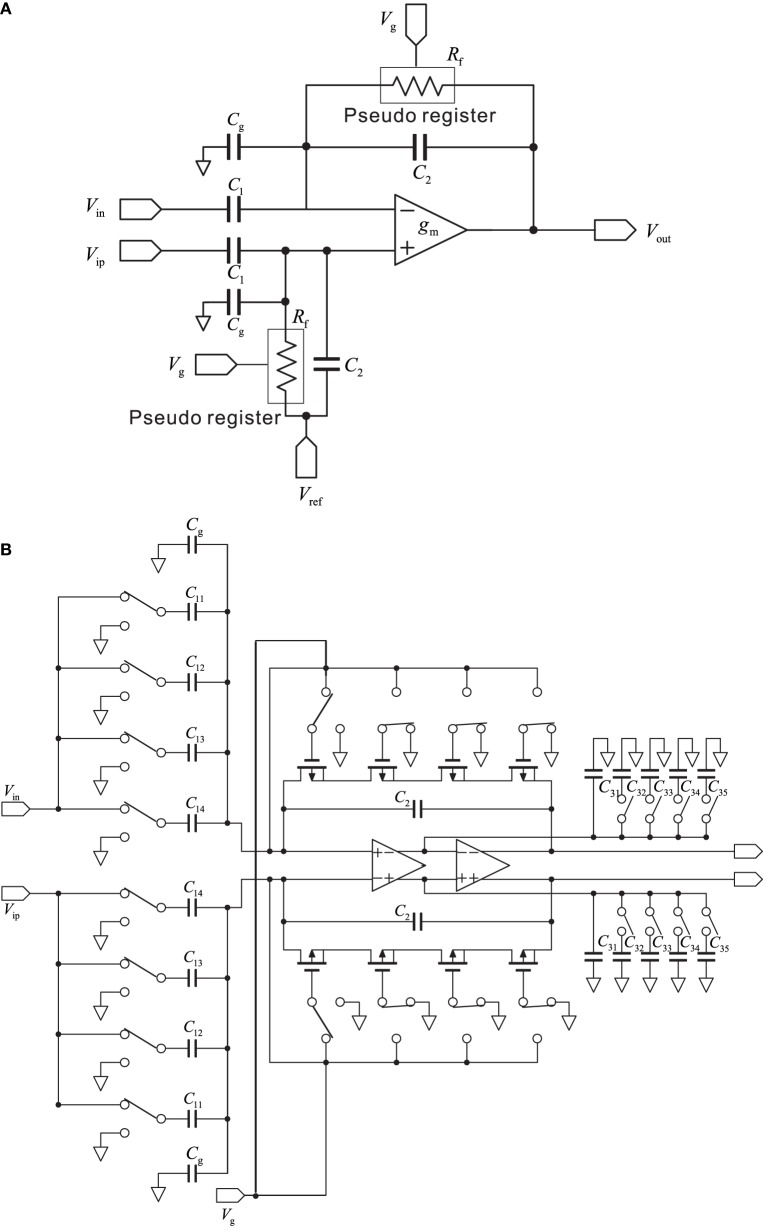
**(A)** Schematic of a standard capacitive-feedback amplifier for neural signal amplification of each channel. The mid-band gain *A*_m_ is set by *C*_1_/*C*_2_, and *g*_m_ is the transconductance of the operational transconductance amplifier (OTA). MOS-bipolar transistors act as pseudoresistors and are represented by simple resistors *R*_f_. Gate voltage of the pseudoregister, stray capacitance, and reference voltage are respectively represented by *V*_g_, *C*_g_, and *V*_ref_. **(B)** Schematic of the low-noise amplifier with variable corner frequencies of low-and high-pass filters (see text for more detail).

In this study, we used a conventional, variable-bandwidth low-noise amplifier (LNA) for the neural recording system (Zou et al., 2009), as shown in Figure 2A. The LNA consists of an OTA, AC-coupled input capacitors *C*_1_, feedback capacitors *C*_2_, and tunable pseudoresistors as feedback resistors *R*_f_. The input capacitors in the circuit that are not directly used are represented as *C*_g_. The corner frequency of the HPF is controlled by the tunable pseudoresistors. The filter cancels drift and offset of the electrode, which can be significantly larger than the signal amplitude. The mid-band voltage gain is set by *C*_1_/*C*_2_, and the lower corner frequency is adjusted by the bandwidth of the OTA and is given by the capacitor *C*_2_ and resistor *R*_f_. The value of *C*_2_ is a trade-off between gain accuracy and corner frequency. To realize a corner frequency of 1 Hz, for example, a large resistance *R*_f_ is required (e.g., *R*_f_ = 3.2 TΩ). Such large resistance has been realized by using a MOS resistor (Harrison and Charles, 2003). The lower corner frequency can be tuned from approximately 1 Hz to about 1 kHz, which corresponds to a resistor with resistances between 32 G and 32 TΩ (Table 1). In this study, the LNA consisted of a fully differential operational amplifier, a buffer amplifier, AC-coupled capacitors *C*_1*i*_ (*i* = 1, …, 4), feedback capacitors *C*_2_ and *C*_3*i*_ (*i* = 1, …, 5), gain and corner-frequency selector switches, and feedback resistors implemented by cascade MOSFETs (Figure 2B). The corner frequency of the LPF was controlled by the switches of the capacitors *C*_3*i*_ (*i* = 2, …, 5). Thus, the LNA realizes high-input impedance and both HPF and LPF characteristics. The parameters illustrated in Figure 2B are listed in Table 1. The details of the amplifier transfer function and the corner frequencies are described in Supplementary Material.

**Table 1:** **Summary of the parameters in the low noise amplifier**.

**Elements**	**Parameters**	**Unit(s)**
*C*_11_	1.00 × 10^−12^	F
*C*_12_	5.00 × 10^−13^	F
*C*_13_	5.00 × 10^−13^	F
*C*_14_	3.00 × 10^−12^	F
*C*_2_	5.00 × 10^−14^	F
*C*_31_	2.70 × 10^−12^	F
*C*_32_	1.00 × 10^−12^	F
*C*_33_	1.90 × 10^−12^	F
*C*_34_	5.80 × 10^−12^	F
*C*_35_	1.67 × 10^−11^	F
*R*_*f*,1_	3.20 × 10^10^	Ω
*R*_*f*,2_	3.20 × 10^11^	Ω
*R*_*f*,3_	3.20 × 10^12^	Ω
*R*_*f*,4_	3.20 × 10^13^	Ω
*g*_*m*_	1.80 × 10^−5^	S

### Stimulation circuitry

In this system, each electrode can be switched between recording and stimulation, and stimulation of neuronal cells can be realized by voltage pulses. The stimulus signal is approximately 500 times the recorded signal or larger, and changes very rapidly. It therefore causes extremely large artifacts, which can totally obscure spikes and saturate the high-gain recording amplifier. To avoid this, the recording inputs should be disconnected during stimulation. However, if the input level of the recording amplifier is floating during stimulation, its output is highly unstable and this will cause additional transients. Thus, the input level should be kept fixed at some value. Our design goal was to fix it at the input level that exists just before the stimulus is applied, which is nonzero because of the DC offset between electrode and electrolyte. This DC offset is also important to consider in generating reliable stimulation of known amplitude. The standard method of electrical stimulation is to apply constant voltage (or current) pulses to the electrode through an isolating circuit. If, however, there is a nonnegligible potential difference between stimulus and reference electrodes, then direct application of the stimulation pulse between electrode and the reference electrode produces an error in the stimulus applied at the interface of the electrode terminal. Therefore, a reasonable design goal is to add the stimulation pulse to the initial offset level (Jimbo et al., 2003).

To reduce stimulus transients, there is one further practical factor that should be considered. The electrode/electrolyte interface has a large capacitive component, which is charged by the electrical stimulation. Because of the slow time constant of the cellular/tissue interface and the series resistance attached to it, it is not discharged rapidly. As a result, the potential difference between stimulation electrode and reference electrode is not identical before and after the stimulus. This causes another transient when the electrode is reconnected to the recording input after stimulation, which can easily be large enough to produce unwanted excitation. Therefore, a further design goal was to include a low-impedance discharge path for the injected electrical charge. To realize this, we previously constructed a system for MEA-based multisite stimulation (Jimbo et al., 2003). However, the design was based on discrete off-chip components and the size was relatively larger than that of an animal brain. In contrast, Heer et al. reported a CMOS metal-electrode-based microchannel system for stimulation and recording with neural cells *in vitro* (Heer et al., 2007). In this study, although our design concept was similar to that of Heer et al. (2007), our goal was to apply it not only to stimulation and recording of neurons *in vitro* but also to those *in vivo* for our future work.

**Figure 3:**
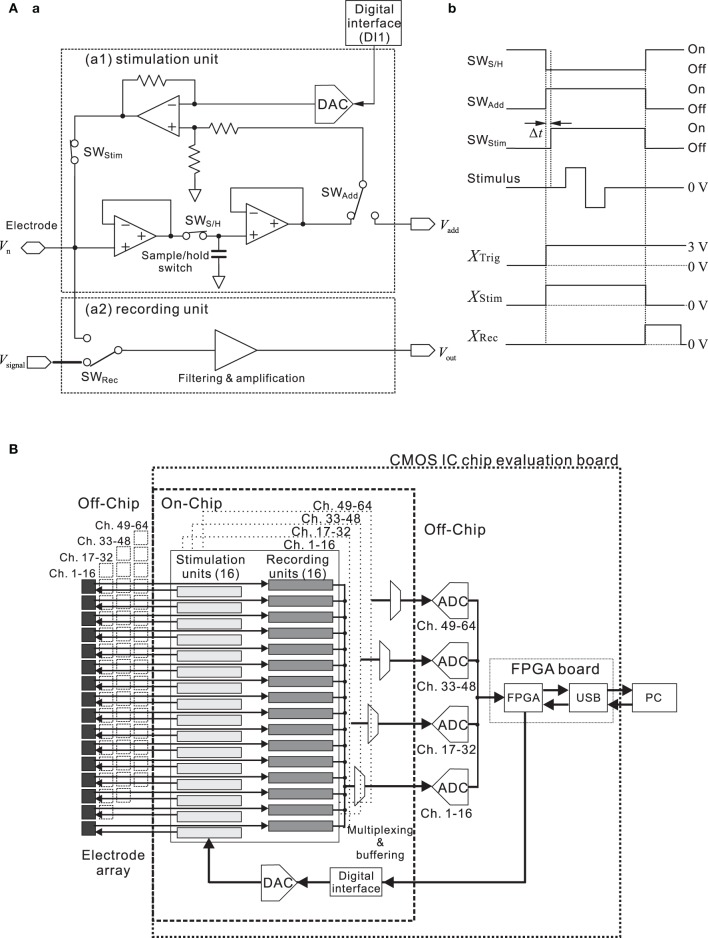
**(A)** Simplified block diagram **(a)** and timing chart **(b)** of a single channel in the interface circuit designed for the stimulation and recording system. The circuit incorporates a preamplifier, logic-driven switches, and a sample-and-hold circuit. Timing of the three switches (SW_S/H_, SW_Add_, and SW_Stim_) and that of the applied stimulus is controlled as shown in **(b)**. To allow for immediate read-out after stimulation, the simulating electrode can be reset and post-stimulation artifacts are reduced on the basis of the circuit and timing. Prior to stimulus application, the amplifier input is disconnected from the electrode by the switch (SW_Rec_) in the recording unit. When SW_Add_ is turned on, the DC offset stored by the capacitor is added to the stimulus voltage in the stimulation mode. During the stimulus pulse, the input of the main amplifier is maintained at the constant level stored in the sample-and-hold circuit. This operation is necessary to minimize the damage to the neural cells. In this system, each switch control signal is triggered by a different signal (*X*_Trig_, *X*_Stim_, and *X*_Rec_), and all are transmitted through the FPGA card from a PC. Before SW_Stim_ turns on, there exists a short time delay Δ*t* after SW_Add_ is turned on. The time delay depends on the stimulation time slot (time divisions of the stimulus pattern) and ranges from 6.25 to 62.5 μ s. In addition, *X*_Trig_ can be replaced with a signal provided by an external trigger source (cf. Figure 1). **(B)** Simplified circuit diagram of the designed system. The CMOS IC chip consists of 64-channel low noise amplifiers (recording units), designed to sense small neural signals, and stimulation signal formation circuits. At the system level, our approach to the multiple sensory channel system consists in sharing a fast digitizer between the multiple channels. That is, 16 recording units are directed toward the analog-to-digital converter (ADC), employing time-division multiplexing in the analog domain.

For one unit of the 64-channel stimulation system, Figure 3A shows the block diagram of the interface circuit. The circuit in the stimulation unit incorporates a voltage amplifier, low charge injection switches, and a sample-and-hold circuit. The timing of the applied stimulus and of the three switches (SW_Stim_, SW_S/H_, and SW_Add_) is controlled, so that the voltage of each electrode before stimulation is fixed (Jimbo et al., 2003). In addition, the stimulus waveforms are created by the waveform generator (DAC) on the CMOS IC chip, according to the information from the DI1 with specific parameters. During recording, the DC offset level at each electrode is continuously monitored and stored in the sample-and-hold circuit (Figure 3Aa).

Before stimulation, SW_Add_ and SW_Stim_ are “Off,” and the DAC value is zero (Figure 3Ab). In this state, therefore, the stimulation circuit is not active and the amplifier processes neuronal signals. Prior to stimulus application, in the first step of a stimulation process, the amplifier gets disconnected from the electrode by setting the record switch (SW_Rec_) to “Off,” and the amplifier input potential is then held at a constant value by a capacitor. When SW_Add_ is turned on, the DC offset (*V*_add_) stored by the capacitor is added to the stimulus voltage in the stimulation mode. Thus, once the amplifier is disconnected, the stimulation signal can be applied to the electrode. In this phase, SW_Stim_ is set to “On” after a short delay (Δ*t*), and the DAC value can be updated, depending on the required stimulation pattern and time slots (i.e., time divisions during a stimulation period for stimulus pattern update). After the stimulus pulse, SW_Stim_ and SW_Add_ are set to “Off,” and the electrode residual voltage is discharged during one time slot. Once the discharging is finished, SW_Rec_ is set to “On,” and the system is back in the sensing (recording) mode. These operations are necessary to minimize the damage to the neural cells and suppress the stimulation artifacts.

To realize multisite stimulation using a small number of input/output (I/O) pads, we designed a serial-to-parallel digital converter and a 5-bit DAC for each of 64 channels (Figure 3B). This circuit system can supply a set of voltage time series to a 64-channel pad connected to each electrode. The digital data converted from the serial digital data are stored in a flip-flop circuit of each channel, and a 64-channel stimulus signal pattern is applied simultaneously at a clock-timing from 1.6 to 16 kHz. As a result, this clock-timing determines the pulse width and stimulation patterns, including interpulse intervals. Thus, this system realizes a programmable stimulus pattern both spatially and temporally. The maximum and minimum stimulation signal voltage amplitude employed with a biphasic/monophasic voltage waveform is ±2.5 V. The reference voltage is created simply with a resistance ladder. Therefore, the peak voltage is tunable. In the stimulation protocol, the mode is first switched from the sensing mode to the stimulation mode, then a reset signal is applied to the serial-to-parallel controller, and finally, continuous serial digital data are converted to an analog voltage waveform for stimulation at any channel with any timing (cf. timing chart in Figure 3Ab). The circuit speed is sufficiently high and the time interval of switching is less than 62.5 μ s; therefore, a waveform generator system controlled with serial digital data can be easily scaled to a 64-channel system.

### Total system circuitry and operation

As mentioned above (Figure 3B), the CMOS IC chip consists of 64-channel LNAs designed to sense small neural signals and stimulation signal formation circuits. This chip makes it possible to realize simultaneous multisite stimulation and neuron sensing through multi-electrodes *in vitro*. Before the operation of the system, parameters of pulse patterns and timings of switching between the sensing mode and the stimulation mode are transferred into the chip from custom-made PC software via the FPAG board. The timing is controlled with an on-chip signal synchronized by the main clock (48 MHz) that is supplied from the FPGA card (SX-Card 6, Prime Systems, Inc., Japan). The transition time from the recording mode to the stimulation mode is about 0.063–1.0 ms, which corresponds to the stimulation time-slot clock frequency at 1.0–16 kHz. When the CMOS IC chip is applied to the MEA measurement system, it takes about 1.2 ms in addition to the switching time to sense the evoked neural spikes because the capacitance at the electrode/electrolyte interface discharges toward the initial state (Jimbo et al., 2003). Thus, evoked spikes after the stimulus offset can be recorded after 1.3–2.2 ms, and 1.3 ms after stimulus offset is the minimum latency for recording neural spikes. The recording unit block for neural signal sensing and the stimulation unit block for handling stimulation signals are integrated on the CMOS IC chip (Figure 3B). After they are sensed by the amplifiers, neural signals from 16 electrodes are multiplexed and transferred into each 16-bit ADC on the CMOS IC chip evaluation board (Figure 1). After that, the four ADCs transfer the digital data to the FPGA card, and the data are subsequently transferred to a PC.

### Chip design and fabrication

**Figure 4:**
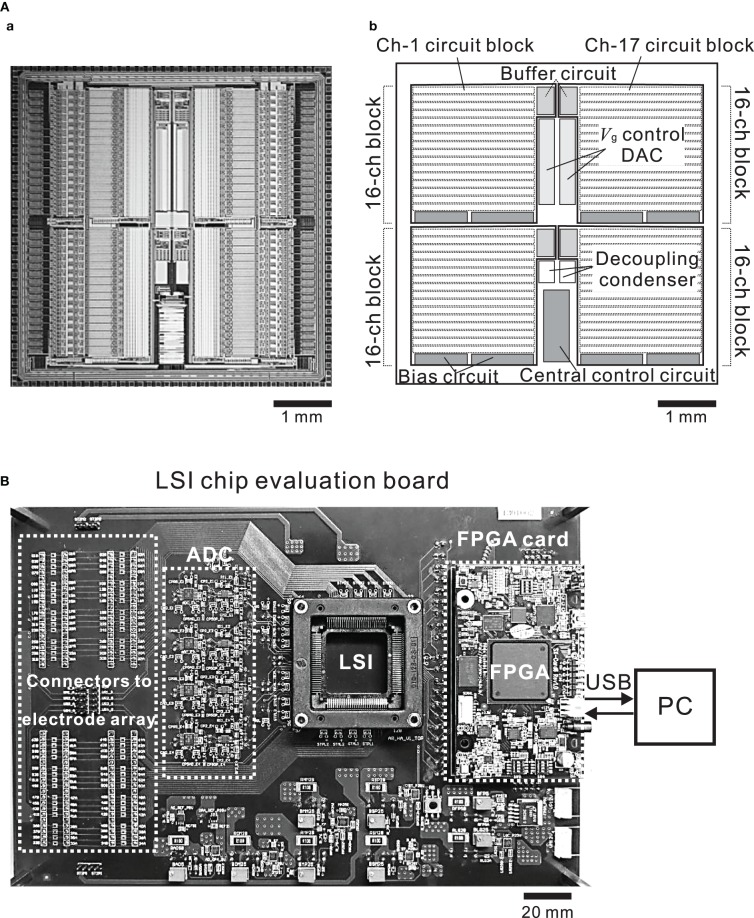
**(A)** Micrograph of the CMOS IC chip **(a)** and schematic **(b)**. The chip comprises 4 buffer circuits, 8 bias circuits, 2 *V*_g_ control ADCs, and 4 recording and stimulation blocks including 16 identical units per block. **(B)** Photograph of the CMOS IC chip evaluation system board. The board includes connectors to an MEA substrate, ADCs, a FPGA card, and an I/O interface to a PC.

The chip was fabricated using industrial 0.25-μ m CMOS mixed-signal technology (TMSC, Taiwan). The micrograph of the 5.0 × 5.0 mm chip (Figure 4A) shows four identical main areas, each of which is assigned 16 channels. Each block in the main areas comprises three sub-blocks; the 16-channel circuit, the buffer circuit, and the bias circuit sub-blocks. The central control circuit is shared by all 64 channels and is located in the bottom central area of the chip (Figure 4Ab). The design of the 16-channel circuit block is modular in that the same circuit unit is repeated 16 times (Figure 4A) and each row of 16 units includes the recording unit of the LNA and the stimulation unit (Figure 3A).

### Functions of FPGA card and CMOS IC chip evaluation board

The large data volumes to be processed and the desired low-latency time require high-performance communication and signal processing capabilities between the CMOS IC chip and a PC. In our system, digital logic components are located on the FPGA on the CMOS IC chip evaluation board and connecting to the CMOS IC chip (Figures 1, 4B). In the two DIs (DI1 and DI2) of the CMOS IC chip, the on-chip digital logic runs at 2.0 MHz and 2.5 V and serves two purposes: (i) operation control in DI1 and (ii) parameter setting in DI2. Firstly, via the operation control DI (DI1), it performs control tasks that include multiplexing, electrode selection for stimulation, and reset of single electrodes, and it also contains the successive-approximation registers of the ADC. Secondly, via the parameter setting DI (DI2), it provides the chip interface to the FPGA. DI2 also stores amplifier settings, including total gain, corner frequencies of LPF and HPF, and pulse pattern stimulation settings. In the CMOS IC chip, these DIs use four lines for the data readout, four lines for parameter settings, two lines for operational control, and four lines for clocks (14 lines in total). All signals are synchronous to the 48-MHz master clock supplied from the FPGA card. In this study, we used a commercially available FPGA card (SX-Card6, Prime Systems Inc., Japan). The card provides the FPGA hardware platform for system designs that demand high performance, serial connectivity, and advanced memory interfacing. It is powered by the Xilinx Spartan-6 FPGA device (XC6SLX75-3FGG676C, Xilinx, USA) and supported by industry-standard peripherals, connectors, and interfaces. Briefly, to manage the data input and output rate of 40 MB/s, the FPGA runs at 48 MHz in conjunction with a USB 2.0 interface chip (CY7C68013A-56LFXC, Cypress, USA) and utilizes 1 GB DDR2 SDRAM (MT47H64M16HR-3, Micron, USA) (see the supplier's web site for more details). The output of the CMOS IC was sampled at 15.6 kHz and 16-bit resolution by the ADCs of the CMOS IC chip evaluation board, and the sampled data were transferred into a PC via the FPGA card. The sampling frequency of 15.6 kHz was the maximum we tested in the experiments below.

### Measuring frequency characteristics of the amplification circuit

To measure frequency characteristics of the chip circuitry, the frequency of sinusoidal signals was controlled between 0.10 and 100,000 Hz using a function generator (33120A, Agilent Technologies Inc., USA) or a frequency response analyzer (FRA 5022, NF Co., Japan). The output signals were analyzed using the frequency response analyzer and a spectrum analyzer (R9211C, Advantest Co., Japan). In addition, to illustrate several analog signal waveforms, the output signals were sampled at 200 kHz using an NI PCI-6259 DAQ card (National Instruments, USA) and custom data acquisition software written in MATLAB R2012 (Mathworks, USA).

### Simulation of circuit models and data analysis for spike detection

An analysis of the designed circuit, including MOS transistors, was performed quantitatively using the Berkeley short-channel IGFET model (BSIM, ver. 4.6.0) simulator (Sheu et al., 1987). In the simulation, MOSFET noise sources included flicker noise, channel thermal noise, induced gate noise, and their correlations. A complete list of the noise model parameters and explanations are described in the user's manual of the BSIM4.6.0 MOSFET model. In this study, a lower or higher corner frequency of a filter transfer function is defined as the frequency at which the filter gain falls by −3.0 dB from the mid-band gain.

To detect neural spikes immediately after stimulation, each recorded voltage transient was first approximated as a *p*-th order polynomial function to minimize the error between the function and the voltage trace. Then, the approximated curve was subtracted from the voltage trace, so that a targeted signal including spikes was obtained. Usually, 5-th to 6-th order polynomial functions were enough to approximate the voltage transients (i.e., *p* = 5 or 6) to avoid overfitting. After the subtraction, spikes were detected when the signal exceeded a threshold. The threshold level for detecting spikes was set at 3.0 times the standard deviation (SD) of the baseline noise (Tateno and Robinson, 2006). The artifact subtraction and spike detection were carried out on off-line in a PC. In all figures, error bars are represented as SDs. By an unpaired *t*-test, a *p*-value less than 0.01 was considered statistically significant. Data are presented as the mean ± SD.

### Rat cortical cell culture

To test our CMOS IC system, we used two *in vitro* preparations: rat cortical cultures and mouse acute brain slices. All animal experiments described below were carried out in accordance with the National Institutes of Health Guidelines for the Care and Use of Laboratory Animals, and with approval of the Institutional Animal Care and Use Committee of Hokkaido University. We used a culture method similar to that described in previous studies (Tateno and Jimbo, 1999; Tateno et al., 2005), with slight modifications. Briefly, cortical tissue of newborn Wistar rats (Japan SLC Inc., Japan) was finely chopped, digested using a papain dissociation system (Worthington Biochemical Co., USA), and mechanically dissociated with trituration. Then, cortical cells were plated on multielectrode substrates (MED-P210A and MED-P515A, Alpha MED Scientific, Japan), coated with laminin (Cat. 354232, BD Biosciences, USA) and poly-D-lysine (P2636, Sigma-Aldrich, USA), and cultured in neurobasal medium (Cat. 21103-049, Life Technologies Co., USA) containing 2% B27 supplement (Cat. 17504044, Life Technologies Co.), 1% GluataMax (Cat. 35050-061, Life Technologies Co.), 2.5 mg/ml insulin (Cat. 10516, Sigma-Aldrich), and 5–40 U/ml penicillin-streptomycin (Cat. P0781, Sigma-Aldrich). The density of cells in the culture was around 15,000 cell/mm^2^. The medium was changed twice a week. All the activity in the cultured networks was recorded *in vitro* from the 30th to the 45th day. Before the measurements with our CMOS-IC based system, the MEAs were tested using commercially available amplifiers (MED-A64HE1 and MED-A64MD1, Alpha MED Scientific Inc., Japan). The material of working and reference electrodes on the MEAs was platinum, and each electrode was covered with platinum black. Contact electrodes for the connectors were also located on the MEA substrate, and the material was indium tin oxide (ITO) with no platinum black. Although during development the electrophysiological properties of cortical neurons change according to the expression of receptors and voltage-dependent ion channels (Luhmann and Prince, 1991), rat cortical neurons previously showed stable patterns of continuous synchronized firing when cultured for the period used in the present study (Kamioka et al., 1996).

### Acute mouse slices including the hippocampus or the auditory cortex

To record FPs from brain slices *in vitro*, transverse or coronal slices (400-μ m thick) including the hippocampus or auditory cortex were prepared from 10- to 15-week-old C57BL/6J and SAMR1 mice (Japan SLC Inc., Japan). In the experimental results presented here, no critical differences between the two mouse strains were found. Details from the electrophysiological experiments will be described elsewhere in the near future. Briefly, chilled artificial cerebrospinal fluid (ACSF) saturated with 95% O_2_ and 5% CO_2_ mixed gas was prepared for use in slicing a brain block including the hippocampus and the cortex. The ACSF contained (in mM) 119 NaCl, 2.5 KCl, 2.5 CaCl_2_, 1.3 MgSO_4_, 1.0 NaH_2_PO_4_, and 11.0 D-glucose (pH = 7.4). A mouse was deeply anesthetized with halothane and decapitated. Then, slices were cut with a tissue slicer (Linear Slicer Pro7, D.S.K., Japan) in the chilled ACSF. The slices were recovered in a submerged-type holding chamber at 28°C in a water bath for at least 2 h before recording. All electrophysiological recording in brain slices was performed with ACSF perfusion and the mixed gas supply from the top of the recording chamber in an incubator (APC-30, Asteck Co., Japan) maintained at 28.0°C. The perfused ACSF solution was also warmed and maintained at 28.0°C, so that the bath was always kept at the same temperature. While recording, slices were plated on multielectrode substrates (MED-P210A and MED-P515A, Alpha MED Scientific, Japan) and covered with a nylon mesh and a stainless slice anchor.

Induction of short- and/or long-term potentiation generally involves short, high-frequency presynaptic stimulation consisting of a series of bipolar pulse trains, termed “tetanic stimulation.” Typical frequencies used to induce such potentiations range 10–250 Hz, and the stimulation contains 20–100 bipolar short pulses. In this particular study, we used a frequency of 125 Hz (i.e., the interpulse interval was 8 ms) and trains of 100 pulses as the tetanic stimulation (TS).

### Acoustic sound and signal processing board

A polyvinylidene difluoride (PVDF) film and a custom-made electric circuit board (Tateno et al., 2013) were used to process acoustic sound and send a trigger signal to the CMOS IC system. The film was used as a piezoelectric sensor to convert acoustic sound pressures into corresponding electric signals (Shintaku et al., 2010). A sinusoidal acoustic wave sound (tone burst) was applied to the sensor device from a speaker (FT96H, Fostex, Japan) located 50 mm away at a 45° angle. To produce a constant sound pressure level (SPL) with a precision of ±0.1 (SD) dB at various frequencies, the speaker was previously calibrated using a measuring amplifier (Type 2636, Brüel & Kjær, UK). The frequency was fixed at 4.9 kHz, which was a resonance frequency of a readout electrode on the film, using a function generator (WF 1973, NF Co., Japan) or a frequency response analyzer (FRA 5022, NF Co., Japan). The duration of the sound waveform was 15 or 20 ms with a 75 dB SPL, and a 4-ms rise/fall time was used. A Hanning window was usually used to obtain a slow onset. For more details about the sensor device and the experimental setup, see Tateno et al. (2013).

## Results

### Circuit frequency response characteristics

**Figure 5:**
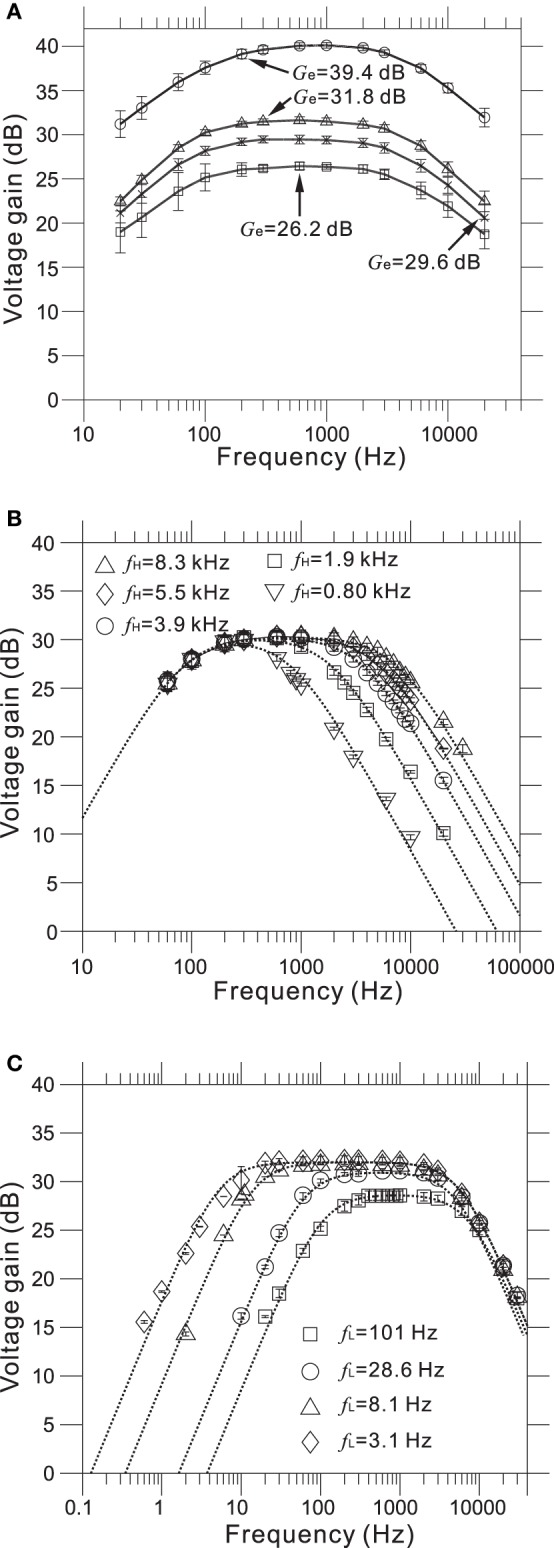
**(A)** Measured frequency response functions (FRFs) of four mid-band voltage gains. The average measured gains (*G*_e_) are 26.2, 29.6, 31.8, and 39.4 dB from smallest to largest (see also Table 2). **(B)** Measured FRFs of five low-pass filters (LPFs) with different higher corner frequencies (HCFs, *f*_H_). The average measured HCFs are 0.80, 1.9, 3.9, 5.5, and 8.3 kHz. The dotted curves represent the corresponding FRFs obtained from approximated transfer functions described in Supplementary Material. **(C)** Measured FRFs of four LPFs with different lower corner frequencies (LCFs, *f*_L_) and the same gate voltage (*V*_g_ = −200 mV). The average measured LCFs are 3.1, 8.1, 28.6, and 101 Hz. The dotted curves represent the FRFs obtained from approximated transfer functions.

Measured transfer functions that characterize frequency response properties of the LNAs and filters we designed are shown in Figure 5. The LNA was designed to give mid-band voltage gains (*G*_d_) of 40.0, 32.0, 29.5, and 26.0 dB via the gain selector switch, and the specific gain could be selected via the DI2 from a PC. For five evaluated chips, the measured mid-band gains (*G*_e_) were 39.4 ± 0.6, 31.8 ± 0.7, 29.6 ± 0.8, and 26.2 ± 0.8 (mean ± SD) from largest to smallest (Figure 5A and Table 2), with the average very similar to that of our design specification.

**Table 2:** **Summary of measured gain and low and high cutoff frequencies in the CMOS IC chip**.

**Gain**	**G_**d**_, Designed at 1 kHz (dB)**	G_**e**_, Measured at 1 kHz (^*^n = ***5***) Mean ± ***SD*** (dB)
	40.0	39.4 ± 0.6
	32.0	31.8 ± 0.7
	29.5	29.6 ± 0.8
	26.0	26.2 ± 0.8
Higher corner frequency (f_H_) of low pass filters	Designed (kHz)	Measured (^*^n = 5) (kHz)
	10	8.25 ± 0.78
	7.5	5.50 ± 0.97
	5.0	3.88 ± 0.62
	2.5	1.89 ± 0.37
	1.0	0.80 ± 0.11
Lower corner frequency (f_L_) of high pass filters	Designed (Hz)	Measured (^*^n = 5) (Hz)
	100	101.3 ± 3.5
	10	28.6 ± 4.3
	1.0	8.1 ± 3.1
	0.1	3.1 ± 3.0

* *n = 5 represents that five amplifiers in different chips were tested*.

As with the amplifier mid-band gain, it is possible to select the higher corner frequencies (HCFs, *f*_H_) of the LPF and the lower corner frequencies (LCFs, *f*_L_) of the HPF (Figures 5B,C). The measured result shows that the tunable HCFs of the LPF were 8.25 ± 0.78, 5.50 ± 0.97, 3.88 ± 0.62, 1.89 ± 0.37, and 0.80 ± 0.11 kHz. Although the proposed amplifier achieved five different HCFs, each measured *f*_H_ was smaller than the corresponding *f*_H_ of the designed LPF (cf. Table 2). For this reason, we consider that parasitic gate capacitances at the OTA output terminals are likely to be responsible for the discrepancy between the designed and measured *f*_H_. Although we could not realize the exact design values of *f*_H_, the ability to adjust the HCFs to target signals can be useful for neural signal recording.

Furthermore, when the gate voltage (*V*_g_) of MOSFETs was set to be −200 mV, Figure 5C shows that the LCFs of the HPF were 101.3 ± 3.5, 28.6 ± 4.3, 8.1 ± 3.1, and 3.1 ± 3.0. However, the designed LCFs were smaller than the measured ones, excluding the LCF of 100 Hz (Table 2). In addition, as dotted lines in Figure 5C indicate the approximated transfer functions of the HPFs, the measured mid-band gains were slightly reduced in a range from 0.5 to 5.0 dB from the designed gain of 32 dB. Because the discrepancy between the measured and designed LCFs of the HPFs is likely to be a variance of *R*_f_, we could not achieve LCFs of under 1 Hz in this chip design and fabrication. Also, we can see that the variance of LCFs in individual chips was relatively larger (cf. Table 2).

**Figure 6:**
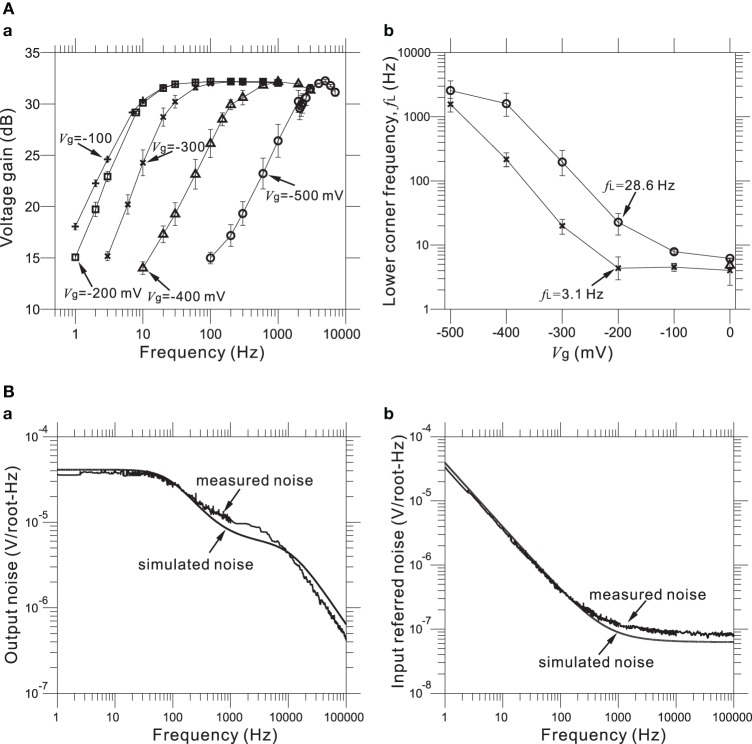
**(A)** In **(a)**, Measured FRFs at 31.8 dB gain with five different values (in mV) of gate voltage *V*_g_ (i.e., from left to right, *V*_g_ = −100, −200, −300, −400, and −500 mV). Excluding the *V*_g_ values, the other parameters of the FRFs are the same as those of the FRF of the HPF, with *f*_L_ = 3.1 Hz and *V*_g_ = −200 mV (Figure 5C). In **(b)**, the relationships between gate voltages *V*_g_ and LCFs of HPFs are shown for two parameter sets (× and °), which respectively correspond to two HPFs with *f*_L_ = 3.1 Hz and *f*_L_ = 28.6 Hz at the identical *V*_g_ = −200 mV (Figure 5C). **(B)** In **(a)**, measured and simulated output-noise spectra for a bandpass filter with a 31.8-dB gain, the smallest LCF (*f*_L_ = 3.1 Hz), the largest HCF (*f*_H_ = 8.3 kHz), and the gate voltage (*V*_g_ = −200 mV) are shown. Similarly, in **(b)**, measured and simulated input-referred noise spectra are shown with the same parameter sets of **(a)**.

Furthermore, values of the gate voltage *V*_g_ profoundly affected the transfer functions, especially those of the HPF. Therefore, an appropriate choice of *V*_g_ value is necessary for the desired transfer function. For five selected values of the adjustable *V*_g_ (e.g., *V*_g_ = −500, −400, −300, −200, and −100 mV) at a mid-band gain of 32 dB, Figure 6Aa shows the frequency responses corresponding to the HPF with a measured LCF of *f*_L_ = 3.1 Hz (Figure 5C) when *V*_g_ = −200 mV. Also, Figure 6Ab shows the dependency of measured *f*_L_ against *V*_g_ for two HPFs, in the two cases of the average measured *f*_L_ = 3.1 and 28.6 Hz for an identical gate voltage (*V*_g_ = −200 mV) in Figure 5C. For the range of *V*_g_ from −500 to 0 mV, we can see that measured *f*_L_ is saturated to around 3 Hz (Figure 6Ab). This result indicates that the value of *f*_L_ ~3 Hz is the smallest LCF of the amplifier, thus limiting the chip design in terms of the LCFs of the HPF. However, in this study, the smallest LCF of around 3 Hz is sufficient for recording local FPs of *in vitro* preparations (see Results below).

In short summary, for each of four 16-channel groups, mid-band gains, HCFs of the LPF, and LCFs of the HPF are adjustable in any combinations for experimental purposes, allowing for selective measurement of specific target signals, including neural spikes, LFPs, and their combination.

For our five LNAs at the measured 31.8 dB gain, Figure 6B shows the average measured output and input-referred noise spectra superimposed with the corresponding spectra of a circuit simulation for a frequency range of 1 Hz to 100 kHz. The circuit simulator used was the Berkeley Short-channel IGFET Model (BSIM4.6.0) (Sheu et al., 1987; see also Materials and Methods). Additionally, the input-referred noise spectra (Figure 6Bb) were obtained by dividing the output noise spectra (Figure 6Ba) by the response function of the amplifier gain. The results demonstrate good agreement between the measured and simulated curves in frequencies below 200 Hz, while a small discrepancy can be seen in the higher frequencies. This indicates that to reduce the higher frequency components, an appropriate selection of LCFs in the HPF is effective for obtaining target signals with a good signal-to-noise (S/N) ratio during the recording of extracellular potentials. For five LNAs, the measured thermal noise level was 93.8 nV / !!HZ!!. Integrating under the area of the measured curve from 10 Hz to 100 kHz (Figure 6Bb) yielded a total input-referred noise of 10.1 ± 0.2 μ V_rms_ (Table 3), while the simulated result was 9.7 μ V_rms_. This is comparable to the thermal noise of the platinum electrodes in physiological saline solution, which was measured to be 6.19 μ V_pp_ for 50 × 50 μ m MEA electrodes in a band between 3.1 Hz and 8.2 kHz in the experiments presented here, because lower and higher frequency components were removed by the bandpass filter. However, the total noise in the actual measurements has been found to be dominated by the actual background activity of the neuronal culture, which in previous studies was often on the order of 10–30 μ V_pp_ in some dense cultures over 30 days *in vitro* (Tateno and Jimbo, 1999; Tateno et al., 2005). Moreover, the measured power consumption of the chip was 1.4 mW at 2.5 V for 64 channels (i.e., 22 μ W/channel), a value relatively higher than those reported in other previous studies (Harrison and Charles, 2003; Hafizovic et al., 2007; Heer et al., 2007). The reason for this could be that the circuit functions not only during recording but also stimulation, for a maximum voltage pulse of ±2.5 V. The obtained results are listed in Table 3.

**Table 3:** **Measured performance summary of the sensor characteristics in the chip**.

**Parameter**	**Measured value**	**Unit(s)**
Process technology	0.25 μm CMOS, mixed signal	–
Number of channels	64	–
Chip size	5.0 × 5.0	mm^2^
Supply voltage	±2.5	V
Total supply current	563	μA
Total power consumption	1.41	mW
Input-referred noise	10.1	μV_rms_
Cross-talk level	−40	dB

### Recording and stimulation in rat cultured cortical neurons

**Figure 7:**
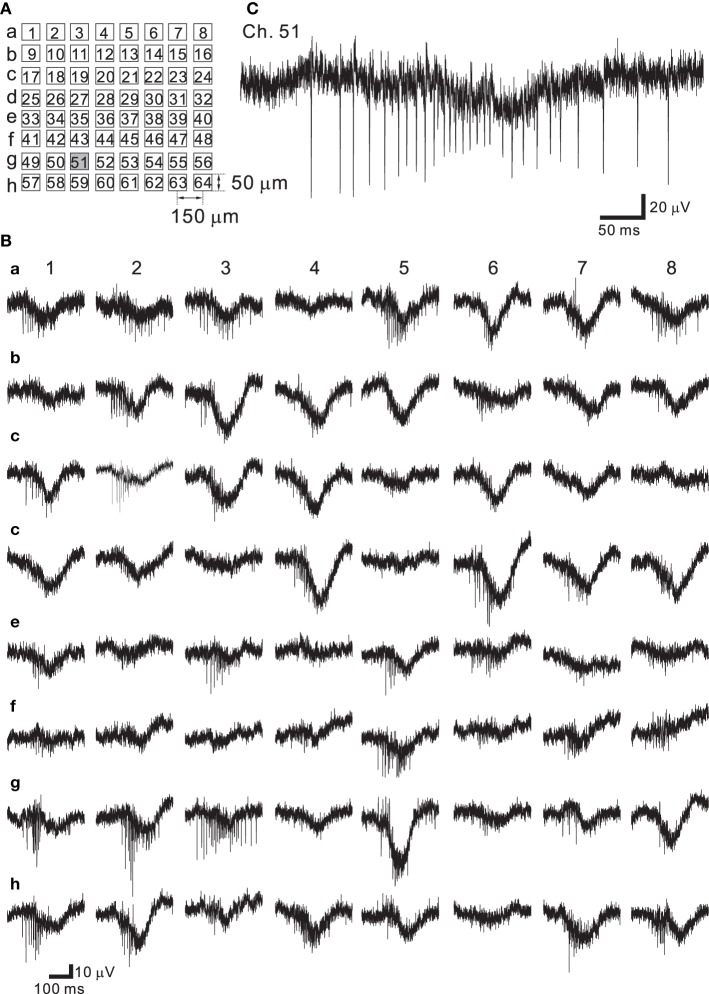
**(A)** Schematic of spatial arrangement of extracellular recording sites on an MEA substrate. The recording sites are labeled as Ch. 1–64 from the top-left corner to the bottom-right corner. **(B)** Extracellular recording of a rat dissociated neuron culture at 37 DIV. Synchronized burst activity occurred at all the recording sites. **(C)** Expanded view at the site of Ch. 51 (gray square in **A**). A fast component (action potentials) and a slow component (slow transient signals with lower frequencies) were observed in the same site.

Neural networks originating from dissociated cortical tissue obtained from newborn rats at postnatal day 1 (P1) were cultured on MEA substrates. During development the electrophysiological properties of cortical neurons change according to the expression of receptors and voltage-dependent ion channels (Luhmann and Prince, 1991; Burgard and Hablitz, 1993). Over a period of around 30 days *in vitro* (DIV), rat cortical neurons cultured on such MEA substrates showed stable patterns of continuous synchronized burst firing (Kamioka et al., 1996; Watanabe et al., 1996). The arrangement of the multielectrodes used in this experiment is shown schematically in Figure 7A. Signals from spontaneously firing neurons after 37 DIV were recorded (Figure 7B). The LCF of the HPF was set to 3.1 Hz and the HCF of the LPF was set to 8.3 kHz. In our measuring system, the choice of the LCF and HFC provided the widest bandwidth in a measureable frequency range. In all 64 recording sites, the signals were successively recorded, and they were mixed signals comprised of a very rapid component (action potentials) and a slow component (FPs with lower frequencies) (Figure 7B). Therefore, the waveform of the signal shows a burst of action potentials superimposed on a slowly changing low-frequency component owing to decreasing FPs in amplitude and subsequent increasing FPs (e.g., see Ch. 51 of Figure 7C). At 37 DIV, the average noise level in these recordings was 18.3 μ V_pp_, which is relatively larger than that (<15 μ V_pp_) reported in our previous studies (Tateno and Jimbo, 1999; Tateno et al., 2005).

**Figure 8:**
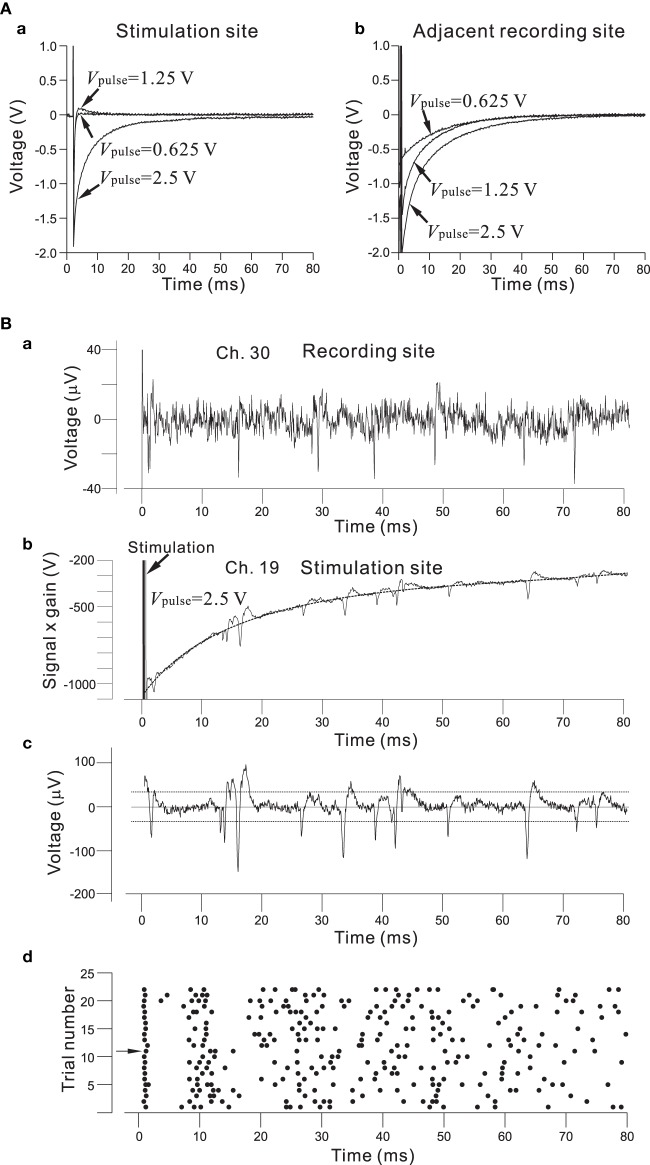
**(A)** In **(a)**, efficiency of the on-chip reset mechanism for stimulation artifact suppression on the stimulation electrode (Ch. 19 in Figure 7A). Bipolar square pulses (150-μ s overall duration) of three different *V*_pulse_ intensities (0.625, 1.25, and 2.50 V) in an ACSF solution have been applied to the electrode. The individual stimulus was anodic first, cathodic second symmetrical biphasic pulses, and each pulse duration was 75-μ s (i.e., 150-μ s overall duration). The fast reset was operational on the electrode for *V*_pulse_ = 0.625 and 1.25 or seemed not to be completely operational for *V*_pulse_ = 2.5 owing to the overload by voltage ranging from −2.4 to +2.4 V. With the reset mechanism, after the offset of stimulation, it takes less than 3 ms for the recording circuitry to return close to baseline in the measuring range. In **(b)**, on the adjacent recording electrodes (Ch. 20 and 150-μ m distance between the two electrodes on the substrate in Figure 7A), because the reset mechanism was not operational on the recording electrode, the voltage traces returned to baseline after a transient period of 20–50 ms. Although the fast reset mechanism was not operational on the adjacent recording sites in the particular experiment, it can be possible. **(B)** In **(a)**, spike events in recording signals from rat cultured cortical neurons at 37 DIV are detectable on a recording electrode (Ch. 30). With this recording filter setting, the gain, LCF, and HCF were 39.4 dB, 28.6 Hz, and 8.3 kHz, respectively. In **(b)**, for *V*_pulse_ = 2.5 V, a transient voltage trace to baseline was obtained from the recording on the stimulation electrode (Ch. 19 in Figure 7A). The transient waveform was approximately fitted by a 5-th order polynomial as a function of time, as indicated by a dotted curve. In **(c)**, a waveform including action potentials is shown after subtracting the polynomial function from the trace in **(b)**. Comparison of the two waveforms shown in **(a)** and **(c)** indicates that action potentials in **(c)** are large enough to detect the event timings. For spike detection threshold, two dotted lines indicate values three times the standard deviation of the baseline noise. In **(d)**, a raster plot shows spike timings (dots) derived from the analysis of data obtained from the stimulation electrode for 22 identical trials. For each trial, the first spike at around 2.5 ms after the stimulation offset occurred with 100% reliability, while subsequent spike events were not reliable.

As described in the Materials and Methods Section, the architecture of the chip allows for the selection of an arbitrary set of electrodes for stimulation, either with individually different stimulus waveforms or an identical stimulus waveform in all 64 channels. The stimulus waveforms consisted of a series of mono/bi-polar pulse trains. Figure 8A shows the efficiency of the on-chip reset mechanism for stimulation artifact suppression. Single bipolar pulses (150-μ s overall duration) of three different intensities *V*_pulse_ (0.625, 1.25, and 2.50 V) in an ACSF solution have been applied to one stimulation electrode (Ch. 19 in Figure 7A), while the fast reset was operational on the electrode for *V*_pulse_ = 0.625 and 1.25 V or seemed not to be completely operational for *V*_pulse_ = 2.5 V owing to overload by voltage ranging from −2.4 to +2.4 V (Figure 8Aa). With the reset mechanism, after the offset of stimulation, it took less than 3 ms for the recording circuitry to return close to baseline in the measuring range. In the same stimulation and recording trial, the simultaneous measurement from the stimulation and adjacent recording electrodes (Ch. 20 and 150-μ m distance between the two electrodes on the substrate in Figure 7A) showed that because the reset mechanism was not operational on the adjacent recording electrode, the voltage traces on the adjacent recording electrode returned to baseline after the transient period of 20–50 ms, whose duration depended on the LCF of the HPF and the value of *V*_pulse_ on the stimulation electrode (Figure 8Ab). However, if the voltage traces obtained from the two electrodes were compared in more detail, the slower reset mechanism was in fact operational on the stimulation electrode for *V*_pulse_ = 2.5 (Figure 8Bb). Therefore, even for the case of *V*_pulse_ = 2.5, using an off-line data analysis that included a subtraction method, action potential events were reliably detectable as shown in Figure 8Bc. The raster plot in Figure 8Bd shows spike timings (dots) derived by analyzing the data obtained from the stimulation electrode over 22 identical trials. For each trial, the first spike at around 2.5 ms after stimulation offset occurred with 100% reliability, while subsequent spike events were not reliable. Note that in our stimulation method as well as that reported in Hafizovic et al. (2007), a stimulation sequence always ends with stimulation of a value close to the equilibrium potential of the electrode, so that the electrode quickly returns to its equilibrium potential before the subsequent stimulation. In addition, to reduce damages to the electrodes and cells, we suggest that electrode materials with lower capacitance than Pt should be better to operate the stimulation artifact suppression mechanism properly.

### Recording and stimulation in mouse hippocampal slices

In the hippocampus, long-term potentiation (LTP) is known to involve activity-dependent persistent changes in synaptic strength. LTP is a multiphasic phenomenon and current models divide it into at least three different phases: initial LTP (I-LTP), early LTP (E-LTP), and late LTP (Frey et al., 1993; Roberson et al., 1996; Sweatt, 1999; L-LTP), although an intermediate LTP phase has also been suggested (Winder et al., 1998). I-LTP represents the first stage of LTP (Kauer et al., 1988; Anwyl et al., 1989; Malenka, 1991) and is further subdivided into post-tetanic potentiation (PTP) for a persistent duration of 0.5-4 min and short-term potentiation (STP) for a duration of 10-15 min (Schulz and Fitzgibbons, 1997). For more details regarding phases of LTP, see two recently published books (Andersen et al., 2007; Sweatt, 2009). To test our CMOS IC system in the stimulation of hippocampal slices, we focused particularly on I-LTP, because I-LTP is the first stage and the most effective for demonstrating the ability of stimulation to induce LTP during short-time in experiments.

The major input to the CA1 area of the hippocampus is from CA3 pyramidal cells via the Schaffer collaterals. Axons of CA3 pyramidal cells heavily innervate both the stratum radiatum and the stratum oriens of the CA1 area. I-LTP of Schaffer collateral fibers in hippocampal area CA1 is defined as the sustained potentiation, with a duration of at least 15 min, that can be induced by a short burst of high-frequency stimulation (HFS). Moreover, on the basis of the time course of decremental phases, HFS can also induce two distinguishable decremental phases (PTP and STP) as stated above (Schulz and Fitzgibbons, 1997).

**Figure 9:**
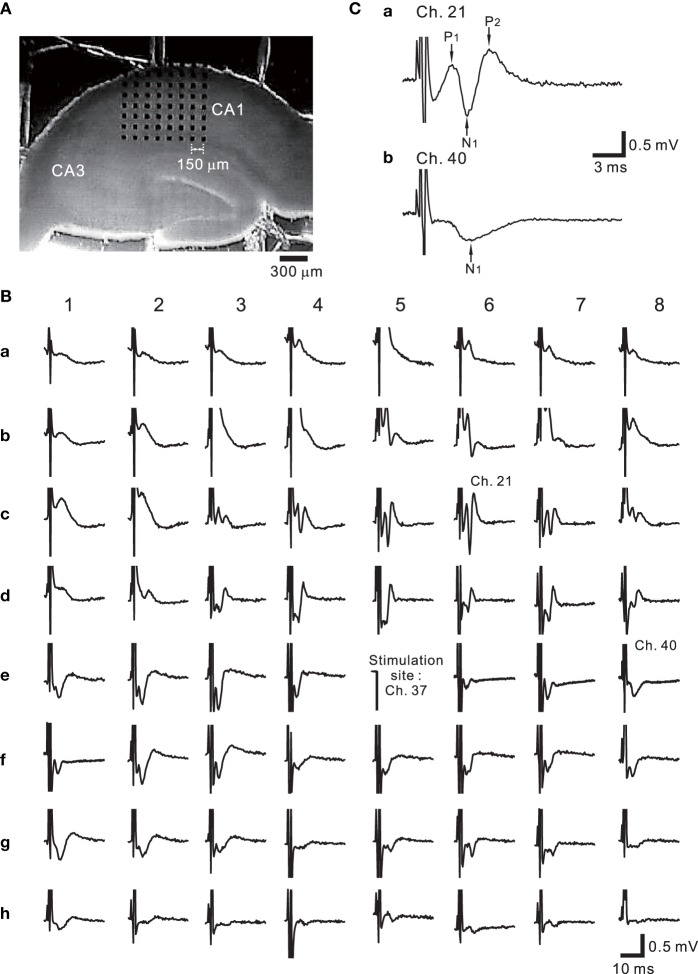
**(A)** One example of hippocampal slice placement on the multielectrode array (MEA) centered in the apical dendritic layer in the stratum radiatum of the CA1. The MEA probe consisted of an array of 64 planar microelectrodes, each 50 × 50 μ m, arranged in an 8 × 8 pattern with a 150-μ m interpolar distance. **(B)** Extracellular recording of field potentials from a mouse slice including the hippocampus. In response to a short bipolar stimulus (±2.5 V) at Ch. 37, field population spikes and excitatory postsynaptic potentials (EPSPs) were evoked on almost all sites excluding the stimulation site. The individual stimulus was anodic first, cathodic second symmetrical biphasic rectangular pulses, and each pulse duration was 100-μ s (i.e., 200-μ s overall duration). **(C)** In response to the stimulation at Ch. 37, the population spike recording at Ch. 21 in the stratum pyramidale of CA1 is shown in **(a)**. Similarly, monophasic population EPSP was recorded at Ch. 40 in the stratum radiatum.

**Figure 10:**
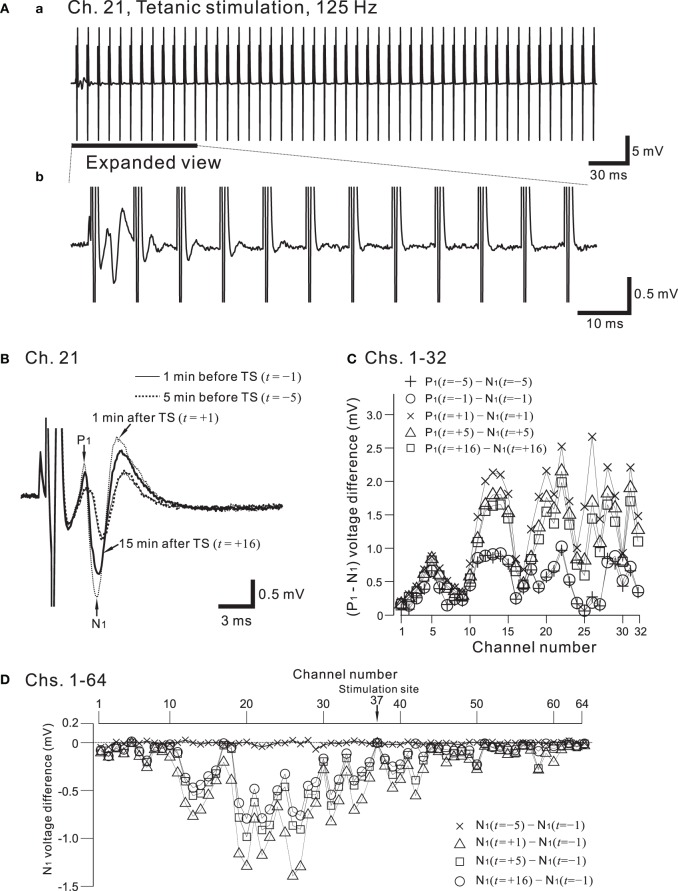
**(A)** In **(a)**, a typical example of recorded field potentials in response to a high-frequency stimulation (HFS), referred to as the tetanic stimulation (TS), at a recording site, Ch. 21 (cf. Figures 7A, 9A). The TS applied at a stimulation site (Ch. 37) consists of 100 repetitions of identical short bipolar pulses with an intensity of 2.5 V at 125 Hz for a duration of 0.8 s. In **(b)**, in response to the same TS shown in **(a)**, the expanded view at the recording site is illustrated for the time interval that includes the first 12 pulses. The evoked responses of the field potential were gradually reduced in amplitude as the pulses were repeatedly applied. **(B)** From the time when the TS was applied (*t* = 0 min), examples of field population spikes at four time points: 5 min before the TS (*t* = −5, thick dotted curve), 1 min before the TS (*t* = −1, thin curve), 1 min after the TS (*t* = +1, thin dotted curve), and 16 min after the TS (*t* = +16, thick curve). After the stimulation offset, the first positive and negative peaks are labeled by P_1_ and N_1_, respectively. **(C)** Voltage differences between P_1_ and N_1_ at Chs. 1-32 for each time point (*t* = −5, −1, +1, +5, and +16 min) to quantify peak intensities of the population spikes and EPSPs at multiple sites during a series of trials. **(D)** On the basis of pre-tetanic negative peak amplitude (*N*_1_(*t* = −1)), in all 64 sites, negative peak voltage differences between two time points are calculated; that is, *N*_1_(*t* = s) − *N*_1_(*t* = −1), where *s* = −5, +1, +5, and +16 min.

Figure 9A shows the placement of a slice on the MEA centered in the apical dendritic layer in the stratum radiatum of the CA1. In this study, for orthodromic stimulation, a stimulating site was usually selected in the stratum radiatum; for example, the electrode labeled as Ch. 37 was the stimulation site in this particular experiment. Extracellular field population excitatory postsynaptic potentials (EPSPs) in all sites, excluding the stimulation site, are shown in Figure 9B. With this placement of the slice on the array, population spikes were recorded on some upper electrodes in the cell body layer of the stratum pyramidale (e.g., Ch. 21 in Figure 9Ca), and monophasic population EPSPs were recorded in the apical dendritic layer of the stratum radiatum (Ch. 40 in the Figure 9Cb). In addition to a usual LTP experiment, a single bipolar pulse (±1.25 or ±2.50 V) was applied per trial at the beginning of a series of three sessions, and several trials were performed as a control session. Next, at time *t* = 0 after the control session, HFS, referred to as the TS, was applied once at 125 Hz with an intensity of 2.5 V for a duration of 0.8 s (recording site Ch. 21 in Figure 10A) at a single site (Ch. 37). Then, single bipolar pulses were repetitively applied as a test session for over 15 min with the same stimulation parameter set as used in the control session. Examples of population spikes from the series of sessions are shown in Figure 10B before and after TS (i.e., at time points of *t* = −5, −1, +1, and +16 min). One minute after TS (*t* = +1 min), amplitudes of the first positive and negative peaks (P_1_ and N_1_ in Figure 10B) increased, but gradually decreased as time passed. For example, the N_1_ amplitude at 16 min after TS (*t* = +16 min) was 27% smaller than that at 1 min after TS (*t* = +1 min), although the amplitude was still larger than those of the control level at 1 and 5 min before TS (*t* = −5 and −1 min). To quantify the peak intensities of both the population spikes and EPSPs at multiple sites during a series of trials, voltage differences between P_1_ and N_1_ were calculated (Figure 10C). The results from over 20 sites indicated that the intensities of the population spikes and EPSPs after TS were increased by over 50% for three slices (*n* = 3). Similarly, on the basis of pre-tetanic negative peak amplitude (N_1_(*t* = −1)), negative peak voltage differences (i.e., N_1_(*t* = +5) −N_1_(*t* = −1)) between two time points were calculated (Figure 10D). At 26 sites, the N_1_ voltage difference showed a significant change (*p* < 0.01). In short summary, these results indicate that our CMOS IC-based multichannel system is capable of inducing I-LTP in mouse hippocampal slices. In particular, the voltage stimulus intensities in these experiments were confirmed to be large enough to induce I-LTP.

### Recording and stimulation in mouse brain slices including the auditory cortex

**Figure 11:**
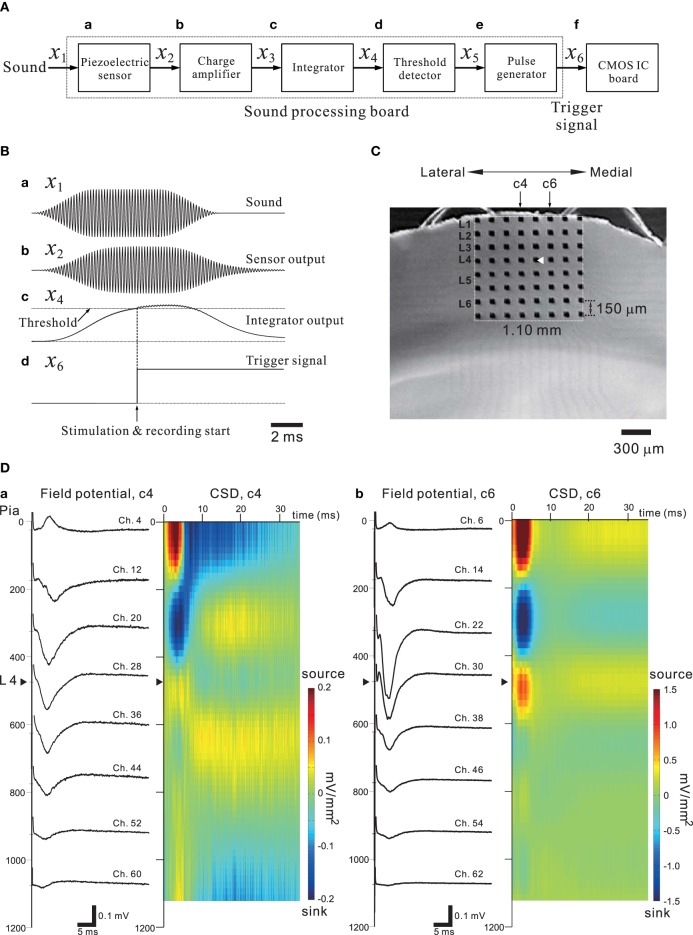
**(A)** A simple block diagram of the acoustic sound processing board (SPB) for mimicking the mammalian peripheral auditory system. A pulse generator to trigger the stimulation and recording processes of the CMOS IC system (CMOS IC board) was incorporated using the output signal of the SPB. Signals on the SPB are processed from left (sound) to right (CMOS IC board). The variables above arrows between two blocks indicate the corresponding signals transmitted from the left to the right block. **(B)** In **(a)**, a transmitted acoustic signal *x*_1_ from a speaker to the sensor is shown. The sound level was 75 dB SPL. Similarly, in **(b–d)**, the waveforms indicate a sensor-output signal *x*_2_, an integrator signal *x*_4_, and a trigger signal *x*_6_ applied to the CMOS IC board, respectively. The threshold level of the threshold detector was 1.4 V, and the output voltage of the pulse generator was 2.5 V. For more details, see Tateno et al. (2013). **(C)** A mouse coronal slice including the primary auditory cortex overlaid on the MEA substrate. The MEA probe is the same as shown Figure 9A. The covered area on the MEA probe of the electrodes is 1.10 × 1.10 mm^2^. The position of the laminar cortical structure is approximately indicated by labels illustrated as layer 1 (L1) to L6. The stimulation site (Ch. 29) was indicated as a white triangle. The individual stimulus was anodic first, cathodic second symmetrical biphasic rectangular pulses, and each pulse duration was 100-μ s (i.e., 200-μ s overall duration). **(D)** Profiles of FPs (left) and CSD (right) are shown for two columns (c4 and c6) of eight electrodes on the left and right sides of the stimulation site (Ch. 29).

In general, sensory information from the thalamus enters the sensory cortex by way of thalamocortical synapses to neurons in cortical layer 4 (L4), and these L4 neurons then transmit the information to neurons in L2 or L3 and then to L5 (for review of the auditory cortex, see Winer et al., 2005; Schreiner and Winer, 2007). Happel et al. analyzed cortical laminar profiles of tone-evoked current source density (CSD) in response to acoustic stimulation of the *in vivo* auditory cortex with pure tones, and revealed characteristic spatiotemporal pattern changes between current source and sink in the CSD profiles (Happel et al., 2010). Using our CMOS IC system and *in vitro* mouse preparations including the auditory cortex, we expected to demonstrate similar CSD profiles observed in the auditory cortex *in vivo*. Evoked FPs in cortical slices were recorded using the 64 MEA channels when a bipolar short pulse was applied to one of the 64 stimulation and recording electrode sites. In addition, to mimic the peripheral auditory system and to trigger the stimulation and recording of the CMOS IC system, a piezoelectric acoustic sensor on a custom-made signal processing board (SPB) was used (for more details, see Materials and Methods and Tateno et al., 2013), as shown in Figure 11A. The SPB transforms acoustic sound (e.g., a tone burst with a specific frequency component in Figure 11Ba) into an electric signal (Figure 11Bb) via the piezoelectric sensor, and the signal is substantially rectified and integrated (Figure 11Bc). Once the integrated signal is increased over a fixed threshold, a trigger signal to start stimulation and recording is applied to the CMOS IC system (Figure 11Bd and the timing chart in Figure 3Ab). A mouse coronal slice including the primary auditory cortex (A1) overlying an MEA is shown in Figure 11C. When the top row of the electrodes was placed on the surface layer (i.e., L1), the fourth row from the top was typically located in L4 or the border between L3 and L4. A short bipolar pulse was then applied to an electrode on the fourth row (e.g., Ch. 29 in Figure 11C); the observed profiles of FPs on two columns (c4 and c6 in Figure 11C) of electrodes are shown in Figure 11D. Around 5 ms after the stimulation offset, the maximum amplitude of the FP trace at the electrodes occurred at the sites (Chs. 28 and 30) adjacent to the stimulation site (Ch. 29), and the activity propagated to the superficial and deeper layers. The propagated negative peak was most increased at the electrode in L3 (the third row from the top), although the stimulation electrode was located on the fourth row from the top. Because similar FP profiles were always observed (*n* = 10 slices) in the same slice configuration on MEAs, the corresponding CSD profiles shows typical source-sink-source triplet patterns (Figure 11D). Hence, the characteristic CSD patterns were reminiscent of the CSD patterns obtained in *in vivo* responses evoked by a pure tone stimulus (Happel et al., 2010). The above result implies that *in vivo* microstimulation of L4 neurons of a specific column in a tonotopic map of A1 can provide similar CSD patterns during intrinsic sound processing in A1 in response to pure-tone stimuli.

## Discussion

In this section, we briefly summarize standard and advanced techniques used in designing a CMOS IC-based multichannel system for recording and stimulation of neural networks (for comprehensive review, see Gosselin, 2011; Demosthenous, 2014) and compare them with the specific system we designed here. Next, we discuss the electrophysiological applications of our system to the three neural preparations *in vitro* and a future plan for a CMOS IC-based prosthesis for the auditory cortex *in vivo*.

### System-level architecture

Front-end neural recording interfaces for multi-electrode systems are typically based on two architectures; analog and digital time-division multiplexing (TDM) (Rodriguez-Perez et al., 2013). In the analog TDM architecture (Hafizovic et al., 2007; Shahrokhi et al., 2010), the analog front-end circuits that amplify and filter the neural signal acquired from each of the electrodes are grouped in an array of channels. Each of these channels comprises an LNA and a bandpass filter, followed by a programmable gain amplifier to maximize the output swing. The analog outputs from all the channels are then multiplexed in the time domain, and they are subsequently converted into digital words by an ADC. The generated time-multiplexed data frames can be either digitally processed to compress them, or sent directly to the output as raw data. In this study, we also employed the analog TDM architecture, and TDM data were stored in a PC through the FPGA card. The analog TDM architecture is likely to have an advantage for an off-chip controller using amplified neural signals in real time for closed-loop control and stimulation (Rolston et al., 2010), although such a controller would be implemented in an on-chip design.

In contrast, in the digital TDM architecture (Gosselin et al., 2009; Seung Bae et al., 2010) the ADC is not shared between the analog channel outputs; rather, an ADC is embedded in each channel. Then a common digital processor manages the digitized signals from the channels, classifies (or reduces) the data, and sends it to the output. In general, this solution requires a larger silicon area than the approach used by the analog TDM architecture. However, the digital TDM architecture has advantages in power consumption owing to the much lower sampling rate requirement at the digitization stage. In addition, the digital TDM architecture has the benefit of being easily scalable by replicating the channels. A very compact circuit implementation for the topology in the digital TDM architecture is proposed in some studies (e.g., Harrison and Charles, 2003), requiring a very small silicon area per channel with standard CMOS process technology. Exploring this implementation could be one of the future directions of our work.

### Low-noise amplifiers

In recording neural activity with planar multielectrodes and penetrating microelectrodes such as the Utah Electrode Array (Rousche and Normann, 1992; Nordhausen et al., 1996) or the Michigan penetrating probes (Wise et al., 1970; Wise, 1986), the recorded neural action potentials often have amplitudes of only a few tens of microvolts, with most energy concentrated between 100 Hz and 6 kHz (Najafi, 1994). Hence, circuits for neural signal amplification must have low noise performance. In addition, front-end neural amplifiers are required to reject electrode offsets or common-mode interference. In the design of such neural amplifiers, two major methods have been used: clock-based and continuous-time techniques (Gosselin, 2011; Demosthenous, 2014). The noise in CMOS transistors is usually dominated by flicker (1/*f*) noise up to relatively high frequencies of the order of several tens of kHz (Nemirovsky et al., 2001). Therefore, various clock-based techniques have been developed to reduce the effects of 1/*f* noise, and the noise reduction technique is based on switched biasing (Klumperink et al., 2000; Min et al., 2012), chopper modulation (Enz et al., 1987; Denison et al., 2007), and autozeroing (Enz and Temes, 1996; Masui et al., 2007). However, the clock-based techniques described above require a clock generation circuit, and thus suffer from potential problems associated with high-frequency interference and clock feedthrough. In addition, high-frequency switching circuits can increase the complexity and power consumption of the design. Hence, in this study, we did not adopt the clock-based technique.

In contrast, continuous-time techniques have been extensively used in the design of neural amplifiers. The conventional circuit in the continuous-time technique is the AC-coupled operational OTA-based neural amplifier with capacitive feedback (Harrison and Charles, 2003), and it is this circuit that we used in this study. As shown in Figure 2A, the circuit is built around a single-stage OTA with CMOS technology. In addition, as described in the Materials and Methods Section, the ratio of capacitors *C*_1_ and *C*_2_ in Figure 2A sets the mid-band gain of the bandpass response (Figure 5A). The input is capacitively coupled through *C*_1_, so that any DC offset from the electrode-tissue interface is removed. To achieve this, *C*_1_ should be made much smaller than the electrode impedance to minimize signal attenuation (cf. Table 1). Hence, we designed an array of capacitors *C*_1_ and realized selectable mid-band gains with either one capacitor or a combination of several (Figure 2B). Moreover, transistors implement MOS pseudoresistors *R*_f_ with an extremely large incremental resistance (≈ 10^10−12^ Ω, cf. Table 1). This allows the LCF of the input HPFs of the AC-coupled stage to be set to a smaller value than several hertz or millihertz. The LCF is set by the product of *C*_2_ and the MOS pseudoresistor *R*_f_ (Figure 2A), although small changes in *R*_f_ cause variance of LCFs in LNAs. Additionally, in our system, the smallest LCF of HPF is around 3 Hz. This limitation prevents the system from properly recording delta frequencies and restricts its applicability to *in vivo* studies targeting at lower rhythms. The limitation is likely to be owing to leakage current of the MOS pseudoresistor. Possible other strategies to overcome this limitation could be to increase values of capacitance *C*_2_ and/or to change the MOS transistor type (i.e., replacement of PMOS transistors with NMOS transistors). The HCF is a function of the load capacitance (*C*_3_ in Figure 2B), the OTA transconductance (*g*_m_), and the mid-band gain (*C*_1_/*C*_2_). Therefore, to adjust the LCFs and HCFs to appropriate values for target signals, we designed arrays of *R*_f_ and *C*_3_ (Figure 2B). As a result, we could successfully record FP signals and single spikes by changing the LCFs and HCFs of the LNA and adjusting the frequency band.

### Artifact cancelation circuit and artifact suppression

To study connectivity of neuronal networks and evoked responses of neural projections, multi-site electrical stimulation is necessary (Wagenaar and Potter, 2004; Heer et al., 2006; Whitson et al., 2006). For example, Wise et al. developed a 3D-electrode array that had separate stimulation and recording probes, and each of the probes featured 64 sites and 8 channels in *in vivo* applications (Wise et al., 2004). Olsson et al. developed thin-film MEAs with integrated circuitry for extracellular neural recording in behaving animals, as well as an eight-site probe for simultaneous neural recording and stimulation (Olsson et al., 2005). In addition, in the context of bidirectional interaction with neural networks, recording neural signals shortly after the stimulation pulse has been applied usually results in stimulation artifacts that mask the neural signals. To reduce these artifacts, Jimbo et al. (2003) for the first time developed an artifact cancelation circuit and incorporated it into a multichannel recording system. In particular, Jimbo et al. presented an MEA-based system where electrodes can be switched between recording and stimulation, using the fast stimulation reset mechanism to record neural signals on stimulating electrodes 3 ms after stimulation (Jimbo et al., 2003). Many CMOS IC-based artifact suppression systems have been developed in recent several studies (Heer et al., 2006; Hafizovic et al., 2007; Hottowy et al., 2008).

In such systems, the stimulation protocol should generally meet two requirements (Jimbo et al., 2003). Firstly, to avoid overloading the amplifier, the input of the recording amplifier has to be disconnected from the electrode during the stimulation period; i.e., there is the capability to perform fast switching from stimulation to recording and vice versa, even on the same electrode (Hafizovic et al., 2007). In the next stage, the amplifier should be connected to a constant potential equal to the electrode DC offset. Secondly, the residual voltage on the electrode should be discharged after the stimulation pulse as quickly as possible. The residual voltage on the electrode after stimulation can generally be minimized by precise balancing of bipolar stimulation pulses. In practice, it is rather difficult to reduce residual voltages to zero, so one must deal with values on the order of several millivolts. Taking into account these considerations, we have developed the circuit shown in Figure 3. In our MEA system, the minimum transition time from the recording mode to the stimulation mode is about 63 μ s. Furthermore, it takes about 1.2 ms in addition to the switching time to sense the evoked neural spikes because the capacitance at the electrode/electrolyte interface discharges toward the initial state (Jimbo et al., 2003). Thus, 1.3 ms after stimulus offset is the minimum latency for recording neural spikes. In combination with off-line data analysis, we can successfully detect spikes within several milliseconds after offset of stimulation (cf. Figure 8) in dissociated cultured neurons, even if the applied voltage intensity is relatively large (e.g., 2.5 V). In contrast, new approaches are needed to record evoked potentials on the stimulation electrodes in the acute slice preparations, because they retuned more slowly to the initial state than those in the cultures (Buitenweg et al., 2000; Braun and Fromherz, 2004; Franks et al., 2005).

### Recording and stimulation of three preparations *in vitro* as a test bench

Cultured cortical neurons were previously found to produce synchronized bursts during their early development (Kamioka et al., 1996; Watanabe et al., 1996). The observed signals from cultured neurons on an MEA substrate were (i) single spikes, (ii) bursts, and (iii) slowly changing FPs with time constants of several hundreds of milliseconds. These signals, each with different frequency components, are all recorded by our CMOS IC-based multichannel system (Figure 7). Although we have not illustrated all examples here, by selecting appropriate LCFs and HCFs we can use this system to filter target neural signals.

The hippocampus plays an indispensable role in many important brain functions, including memory formation and spatial navigation. Also, it is very well suited for slice studies owing to the planar organization of projections among hippocampal subfields. Therefore, a number of researchers have pursued the study of the hippocampus with MEAs (Novak and Wheeler, 1988; Egert et al., 1998; Shimono et al., 2000; Chong et al., 2011). Although these experiments could be performed with traditional instrumentation and a conventional MEA-based multichannel system, we intended to extend the range of such recording systems to our CMOS IC-based multichannel system. Because there is increased throughput owing to the ability to stimulate and record from multiple sites, the different responses expected in the various regions act as within-slice controls that help validate the viability of the slice and the experimental conditions. In this study, by applying a short burst of HFS to Schaffer collateral fibers in hippocampal area CA1, we could induce sustained postsynaptic potentiation with a duration of over 15 min. These results indicate that the stimulation intensity of our system was large enough to induce sustained changes in mouse hippocampal slices.

To test the validity of our system, we applied voltage stimulation to acute mouse slices including the auditory cortex. The stimulation intensities were large enough to evoke large local FPs ranging from several hundred microvolts to several millivolts (Figure 11), so that the obtained responses were suitable for further analysis, for example CSD analysis. In this study, CSD analysis results were reminiscent of source-sink-source triplet CSD profiles from *in vivo* recording in the auditory cortex in response to an acoustic sound stimulus. In addition, to trigger electrical stimulation and recording, we connected the acoustic piezoelectric sensor to the CMOS IC-based multichannel system. The entire system structure we developed can serve as the basis for a neural prosthesis for the auditory cortex *in vivo*. To realize this goal in the near future, the method of converting sound into a stimulation pattern in the brain tissue *in vivo* is the next important target of our study.

An additional related challenge is to design high-resolution CMOS IC-based microelectrode systems that can provide bidirectional access to several individual cortical neurons *in vivo* via electrical stimulation and neural recording capabilities in the same device. Such systems will make it possible to interact with groups of neurons in a closed loop (Rolston et al., 2010); i.e., (i) to make use of electrical stimulation in conjunction with neural recording to activate specific neural networks, (ii) to obtain instantaneous feedback information on their status, (iii) and to electrically modify the activity of the networks as well as the device system parameters. Developing such CMOS IC-based bidirectional neural interfaces will have a profound impact on experimental neuroscience and on the implementation of new assistive technologies to treat chronic diseases and neural disorders, including tinnitus, epilepsy, and Parkinson disease.

## Conclusions

We describe an ASIC system designed for multisite electrical stimulation of neural tissue using MEAs. Our design is intended for applications in systems requiring simultaneous stimulation and recording of signals from various types of neural tissue, both *in vitro* and *in vivo*. The developed ASIC comprises 64 independent stimulation channels, any subset of which may be stimulated, and can generate almost arbitrarily defined bipolar voltage pulses below an amplitude of 2.5 V with 5-bit resolution. Each channel is also equipped with a stimulation artifact suppressor controlled in real time, which reduces the dead time of the system after each stimulation pulse. The system we developed can be used for conventional electrophysiological experiments *in vitro*, and the technology is also a foundation of future auditory neural prostheses applied to the auditory CNS.

### Conflict of interest statement

The authors declare that the research was conducted in the absence of any commercial or financial relationships that could be construed as a potential conflict of interest.
