# Supplementary material for: A CMOS IC-based multisite measuring system for stimulation and recording in neural preparations in vitro
Source: Front Neuroeng. 2014 Oct 10;7:39. doi: 10.3389/fneng.2014.00039 (PMC4193337; doi:10.3389/fneng.2014.00039)
Supplement: Supplementary file 1 [file DataSheet1.DOCX]

**Appendix A**

According to a model proposed by Wattanapanitch and Sarpeshkar (2007), the transfer function *H*(*s*) of the amplifier (Fig. 2A) is modeled as

$H(s)=-\frac{C_{1}}{C_{2}}\cdot\frac{sR_{f}C_{2}}{1+sR_{f}C_{2}}\cdot\frac{g_{m}}{g_{m}+sG_{m}C_{3}}$, (A1)

where *G_m_*=*C*_1_/*C*_2_ and *C*_3_ is the total capacitance of effective capacitors in *C*_3_*_i_* (*i*=1, …, 5) in Fig. 2B. In the above model, the mid-band gain of the amplifier is denoted by *G_m_*. In addition, the lower corner frequency is at *f*_L_=1/(2π*R_f_C*_2_), whereas the higher corner frequency is at *f*_H_=*g_m_*/(2π*G_m_C*_3_). We can relate the input-referred noise $\overline{v_{n}^{2}}$ of the gain-stage OTA to the input-referred noise $\overline{v_{n,\text{amp}}^{2}}$ of the overall amplifier as

$\overline{v_{n,\text{amp}}^{2}}=\left(\frac{C_{1}+C_{2}+C_{g}}{C_{1}}\right)^{2}\cdot\overline{v_{n}^{2}}$, (A2)

where *C_g_* is the parasitic gate capacitance at input terminals of the OTA (Fig. 2A). It is known that the input-referred noise of the gain-stage OTA is the major component of the total noise ([Wattanapanitch et al., 2007](#_ENREF_71)).
